# Correlation between Glycation-Related Biomarkers and Quality of Life in the General Japanese Population: The Iwaki Cross-Sectional Research Study

**DOI:** 10.3390/ijerph19159391

**Published:** 2022-07-31

**Authors:** Masaya Tsubokawa, Miyuki Nishimura, Koichi Murashita, Takuro Iwane, Yoshinori Tamada

**Affiliations:** 1 Innovation Center for Health Promotion, Graduate School of Medicine, Hirosaki University, Hirosaki 036-8562, Japan; y.tamada@hirosaki-u.ac.jp; 2 Health Science Research Center, FANCL Research Institute, Yokohama 244-0806, Japan; nishimura_miyuki@fancl.co.jp; 3 Center of Innovation Research Initiatives Organization, Hirosaki University, Hirosaki 036-8562, Japan; murasita@hirosaki-u.ac.jp (K.M.); i-tak@hirosaki-u.ac.jp (T.I.)

**Keywords:** quality of life, diabetes, glycation, HbA1c, glycoalbumin, pentosidine

## Abstract

The correlation between diabetes-related biomarkers and quality of life (QOL) remains unclear. In this cross-sectional study, we investigated the correlation between diabetes-related biomarkers and QOL in a general Japanese population who underwent health checkups as a part of the Iwaki Health Promotion Project. Male and female participants aged ≥ 20 years from Iwaki District, Hirosaki City, Aomori Prefecture who participated in the 2019 medical evaluation were recruited. QOL was evaluated using the Short Form Health Survey 36 (SF-36). Fasting blood glucose, homeostatic model assessment-estimated insulin resistance (HOMA-IR), hemoglobin A1c (HbA1c), glycoalbumin, and plasma pentosidine were also evaluated as diabetes-related markers. Of the 1065 recruited participants, 1053 completed the clinical and QOL evaluations. Multivariate regression analysis revealed that upregulated diabetes-related markers levels were correlated with decreased SF-36 scores. Blood glucose, HOMA-IR, HbA1c, glycoalbumin, and plasma pentosidine levels were correlated with general health. Moreover, plasma pentosidine levels were correlated with role physical, social functioning, and role emotional in addition to general health. These results indicated that the levels of diabetes-related biomarkers, particularly the levels of plasma pentosidine, a glycation marker, were associated with QOL in our cohort.

## 1. Introduction

Quality of life (QOL), a multidimensional concept, corresponds to the general well-being status of individuals [1] and is an important measure of overall health [2]. QOL represents the goal of all health interventions and is assessed in terms of physical and social function along with mental well-being [3]. Studies on QOL involve various patient groups and research designs [4]. Traditionally, the principal endpoints in medical and health research have been biomedical outcomes rather than QOL outcomes. However, research over the past few decades focused on the QOL of patients, which increased the application of QOL assessments [5]. Increased human life expectancy shifted the research focus to improving the QOL and maintaining good overall health [6]. Leading global studies on health care have emphasized the importance of QOL [7].

The incidence of diabetes mellitus has increased with the aging population, which, due to the required the treatment, has created a major burden on health care systems [8]. As a result, patients with diabetes have low QOL [3]. The goal of early diagnosis and treatment of diabetes is to improve QOL [9] since the progression of diabetes affects the QOL [10]; therefore, diabetes prevention strategies are essential [11]. However, studies on the general population, including healthy individuals, are limited to the correlation between hyperglycemia and depressive symptoms [12]. In addition, complications such as coronary artery disease, renal failure, blindness, and microvascular and macrovascular disease are the major factors affecting the QOL of patients with diabetes [9]. The formation and accumulation of glycation end product (AGE) represent one of the main pathogenic mechanisms underlying diabetes-associated complications [13], although the relationship between AGE and QOL remains to be fully investigated.

Therefore, this study investigated the correlation between several diabetes-related biomarkers, including glycation markers associated with AGEs, and various QOL-related parameters in the general Japanese population who underwent health examinations as part of the Iwaki City Health Examination Project.

## 2. Participants and Methods

### 2.1. Participants and Analysis

Medical examination under the Iwaki Health Promotion Project has taken place annually since 2005 to prevent lifestyle-related diseases, maintain and promote public health of the residents in the Iwaki District of Hirosaki City [14]. Male and female participants aged ≥ 20 years residing in Iwaki District, Hirosaki City, Aomori Prefecture who participated in the 2019 medical examination were recruited. This cross-sectional study recruited 1065 participants, of which 1053 participants completed the clinical examination and QOL evaluation and were therefore enrolled. The study protocol was approved by the Ethics Review Board of the Hirosaki University School of Medicine (approval numbers: 2019-009) and conducted according to the principles of the Declaration of Helsinki. Written informed consent was obtained from all participants [15].

### 2.2. Clinical Features

All laboratory tests were performed under fasting conditions in the morning [15]; body mass index (BMI; calculated using BMI = weight (kg)/height (m)^2^) was assessed during physical examination. The systolic and diastolic blood pressure were recorded using an automated sphygmomanometer Elemano2 (Terumo Co., Ltd., Tokyo, Japan) while the participants were in a seated position. The following hematological parameters were analyzed: the glucose metabolic capacity-related parameters of blood glucose levels and homeostasis model assessment-estimated insulin resistance (HOMA-IR) values; the glycation-related parameters hemoglobin A1c (HbA1c), glycoalbumin, and plasma pentosidine levels; the lipid metabolic capacity-related parameters triglyceride, total cholesterol, high-density lipoprotein, and low-density lipoprotein levels; the liver function-related parameters alanine transaminase, aspartate transaminase, and γ-glutamyl transferase; the renal function-related parameters creatinine and urea nitrogen levels. Blood samples were collected from the peripheral veins with the participants in the supine position and tested by LSI Medience Co., Ltd. (Tokyo, Japan) [15]. To evaluate lifestyle-related parameters, the results of the survey regarding diabetes mellitus, dyslipidemia, presence of hypertension, smoking habit, alcohol consumption, and exercise (non-winter and winter seasons) were also analyzed.

### 2.3. Diabetes-Related Biomarkers

Blood glucose, HbA1c, and glycoalbumin levels were determined using enzymatic methods with JCA-BM9130 and JCA-BM8060 (Japan Electron Optics Laboratory Co., Ltd., Tokyo, Japan). Plasma pentosidine levels were determined using high-performance liquid chromatography with Acquity UPLC H-CLASS (Nihon Waters Co., Ltd., Tokyo, Japan), while insulin levels were determined using chemiluminescent immunoassay methods with Architect i2000 SR (Abbott Japan LLC., Tokyo, Japan). HOMA-IR was calculated as follows: HOMA-IR = (blood glucose levels × blood insulin levels)/405 [16,17].

### 2.4. QOL Evaluation

The Short Form Health Survey 36 (SF-36) is structured based on a universal concept of health and is not limited to a specific disease, and hence can be used to measure the QOL of patients with various diseases along with healthy subjects. QOL can be comparatively analyzed among patients with different diseases. Additionally, the health of patients can be compared with that of the general population.

SF-36 comprises multiple questions to measure the following eight health parameters: physical functioning, role physical, bodily pain, general health, vitality, social functioning, role emotional, and mental health [18,19].

The SF-36 score for each parameter is interpreted as follows:

Physical functioning

Low score: Difficulty with performing routine activities, such as bathing or dressing.

High score: Can perform all types of activities, including strenuous activities.

Role physical

Low score: Difficulties with working or performing regular activities in the last month due to physical limitations.

High score: No difficulties with working or performing routine activities in the last month due to physical limitations.

Bodily pain

Low score: Routine work markedly hampered by severe pain in the last month.

High score: No physical pain in the last month that would interfere with routine work.

General health

Low score: Poor health that gradually worsened.

High score: Good health.

Vitality

Low score: Tiredness and exhaustion at any time during the last month.

High score: Feeling energetic at all times during the last month.

Social functioning

Low score: Difficulty socializing with family, friends, neighbors, or peers in the last month for physical or psychological reasons.

High score: Physical or psychological reasons did not affect the ability to socialize with family, friends, neighbors, or peers in the last month.

Role emotional

Low score: Difficulty with working or performing routine activities in the last month due to psychological reasons.

High score: Psychological reasons did not interfere with working or performing routine activities in the last month.

Mental health

Low score: Nervous and depressed mood at all times during the last month.

High score: Calm, happy, and relaxed feeling during the last month.

### 2.5. Statistical Analysis

Continuous variables among the clinical characteristics of the participants are represented as the mean ± standard deviation, whereas the categorical variables are represented as the sample size and percentage (%). To investigate diabetes biomarkers associated with QOL, univariate regression analysis was performed using the SF-36 scores as the dependent variable and each diabetes-related biomarker as the independent variables. In the multivariate analysis, model 1 (adjusted for age, sex, and BMI) and model 2 (adjusted for antihypertensive medications, exercise, smoking, and alcohol consumption in addition to age, sex, and BMI) were performed. In addition, we performed multivariate regression analysis using SF-36 scores as the dependent variable and age, sex, and BMI as the independent variables. The regression coefficient (β) was calculated using the standard least squares method. In these analyses, the significance level was set at *p* < 0.05 in two-tailed tests, and 95% confidence interval (CI) for the regression coefficient (β) was calculated. We used JMP^®^ Ver.16 software (SAS Institute, Inc., Cary, NC, USA) in these statistical analyses [15].

## 3. Results

Table 1 lists the clinical characteristics of our study cohort. The characteristics of the study cohort were as follows: sex distribution with 59.2% females and 40.8% males; mean age, 52.6 years; mean BMI, 23.0 kg/m^2^. For the SF-36 scores, the vitality and general health were relatively low. The rates of lifestyle-related diseases were highest in high blood pressure followed by hyperlipidemia and diabetes.

Table 2, Table 3, Table 4, Table 5 and Table 6 show the results of univariate and multivariate regression analyses of the association between SF-36 scores and diabetes-related biomarkers.

A multivariate regression analysis (Model 2) adjusted for age, sex, BMI, antihypertensive medication, exercise (non-winter and winter seasons), smoking, and alcohol consumption revealed that general health was negatively correlated with upregulated blood glucose levels (β = −0.081; 95% CI = −0.154 to −0.008; *p* = 0.030), HOMA-IR values (β = −2.646; 95% CI = −4.181 to −1.111; *p* = 0.001), HbA1c levels (β = −3.321; 95% CI = −5.184 to −1.458; *p* < 0.001), glycoalbumin levels (β = −0.883; 95% CI = −1.453 to −0.313; *p* = 0.002), and plasma pentosidine levels (β = −0.093; 95% CI = −0.164 to −0.021; *p* = 0.011). Plasma pentosidine levels were negatively correlated with role physical (β = −0.112; 95% CI = −0.182 to −0.042; *p* = 0.002), social functioning (β = −0.073; 95% CI = −0.140 to −0.006; *p* = 0.033), and role emotional (β = −0.073; 95% CI = −0.144 to −0.001; *p* = 0.048) in addition to general health.

A multivariate regression analysis (Model 2) also showed that levels of diabetes biomarkers such as blood glucose, HOMA-IR, HbA1c, glycoalbumin, and plasma pentosidine were associated with the general health of the SF-36 scores. The *p*-value, which indicates significance, was the smallest for HbA1c, followed by HOMA-IR, glycoalbumin, plasma pentosidine, and blood glucose. Moreover, the level of the glycation marker plasma pentosidine was associated with lower SF-36 scores such as role physical, social functioning, and role emotional compared with other markers.

Table S1 shows the results of multivariate regression analysis when SF-36 scores were used as the dependent variable, while age, sex, and BMI were used as the independent variables. Age was negatively correlated with physical functioning, role physical, bodily pain, general health, and role emotional. Female was negatively correlated with physical functioning, role physical, bodily pain, general health, vitality, social functioning, and mental health. BMI was negatively correlated with physical functioning, bodily pain, general health, and social functioning.

## 4. Discussion

Among the general Japanese population who participated in the Iwaki Health Promotion Project medical examination, blood glucose, HOMA-IR, HbA1c, glycoalbumin, and plasma pentosidine levels were correlated with some SF-36 scores after adjusting for age, sex, BMI, antihypertensive medication, exercise (non-winter and winter seasons), smoking, and alcohol consumption. Therefore, the levels of diabetes-related biomarkers were associated with QOL in our cohort. Particularly, the level of plasma pentosidine, a glycation marker, was associated with QOL. The correlation between QOL and plasma pentosidine levels was not previously reported.

Diabetes markedly deteriorates the QOL of patients to varying degrees [20,21,22,23]. Previous studies compared the QOL of patients with type 2 diabetes with that of healthy subjects. In contrast, we examined the correlation between QOL and diabetes-related biomarkers and found that the levels of diabetes biomarkers such as blood glucose, HOMA-IR, HbA1c, glycoalbumin, and plasma pentosidine were associated with the general health of the SF-36 scores. In addition, poorer general health was associated with aging and obesity along with diabetes-related markers. General health is impaired by the deterioration of the glucose metabolic capacity, which is associated with aging and obesity. Hyperglycemia may contributes to decreasing QOL such as exacerbating symptoms of fatigue and depression [24,25] by modulating physiological pathways (including inflammatory processes) and downregulating neurotrophic functions [26,27,28,29]. Additionally, previous studies demonstrated that individuals with low-QOL-associated anxiety are at high risk for developing diabetes [30]. Therefore, to improve QOL, it is important to educate the general population regarding the high risk of low QOL among individuals in a prediabetic state.

Furthermore, plasma pentosidine levels were negatively correlated with SF-36 scores such as role physical, social functioning, and role emotional compared with other diabetes-related markers. Deterioration in role physical and role emotional were associated with aging. Worsening social functioning was correlated with high BMI, while worsening role physical and role emotional were not. Hyperglycemia is associated with obesity, while plasma pentosidine levels are inversely correlated with BMI [31]. Indeed, high plasma pentosidine levels may lower QOL through an obesity-independent mechanism. Plasma pentosidine is a type of AGE associated with diabetes [32,33]. AGE accumulation can induce inflammation and aberrant signaling in cells, thereby impairing neurological functions [34,35]. Similarly to hyperglycemia, upregulated plasma pentosidine-induced inflammation and neurological function impairment may adversely affect QOL. Moreover, pentosidine is a cross-linking AGE induced by the oxidation of bone collagen cross-links [36]. Serum pentosidine levels are negatively correlated with muscle mass in menopausal women with type 2 diabetes [37]. Additionally, upregulated levels of serum carboxymethyl-lysine, another AGE product, was negatively correlated with walking ability in elderly women [38]; this effect is, among others, attributed to the increased number of AGE receptors in the muscle tissue with age and AGE-mediated inhibition of muscle synthesis [39]. This suggests that pentosidine is associated with both bone quality and physical function [40]. In addition to inhibiting the upregulation of blood glucose levels, strategies that decrease plasma pentosidine levels can reduce the risk of physical limitations.

Plasma pentosidine is also a renal function marker [32,33]. Sarcopenia, characterized by the progressive age-related loss of muscle mass coupled with decreased muscle function, is prevalent among patients with chronic kidney disease (CKD) [41]. The unfavorable outcomes of sarcopenia have attracted attention [42]; sarcopenia-associated loss of skeletal muscle has significant consequences in CKD even prior to initiating dialysis therapy [43]. CKD-associated sarcopenia results from an altered balance between catabolic and anabolic processes that regulate muscle homeostasis [44]. The regulation of homeostasis is a complex process involving hormonal and immunological factors, progenitor cell function, mitochondrial dysfunction, inflammation, metabolic acidosis, malnutrition, physical inactivity, angiotensin II upregulation, and growth factors [44]. These components vary according to the progression of CKD [44]. As sarcopenia and low muscle strength are associated with a poor QOL [45,46], renal function may influence the negative correlation between QOL and plasma pentosidine levels, although the underlying mechanisms remain to be determined.

It was suggested that digital solutions should be developed to fully support the psychophysical well-being of patients with diabetes mellitus [47]. Furthermore, our results suggested that a self-management system and intervention method for glycation prevention can help to improve QOL in the general population.

This study has some limitations. As this was a cross-sectional study, the causal relationship between the factors associated with the decline in QOL could not be determined. The correlation between decreased QOL and plasma pentosidine levels has not been previously assessed. We included individuals who voluntarily participated in the Iwaki Health Promotion Medical Examination Project [15]; thus, the results of this study cannot be generalized due to selection bias. Additional studies are warranted to clarify the relationship between decreased QOL and glycation biomarkers, particularly plasma pentosidine, in the general population using longitudinal data from a large cohort.

## 5. Conclusions

In the general Japanese population, levels of diabetes-related biomarkers such as blood glucose, HOMA-IR, HbA1c, glycoalbumin, and plasma pentosidine were associated with SF-36 scores in the study cohort. Particularly, the levels of the glycation marker plasma pentosidine were associated with lower SF-36 scores compared to other diabetes-related markers. Our results suggest that the QOL of the general population can be improved by preventing glycation in addition to controlling blood glucose levels.

## Figures and Tables

**Table 1:** Clinical characteristics of the study cohort.

		Total	(n = 1053)	Males	(n = 430)	Females	(n = 623)
Continuous Variables	(Unit)	Mean	(SD)	Mean	(SD)	Mean	(SD)
Age	(years)	52.6	(15.24)	52.4	(15.03)	52.7	(15.40)
BMI	(kg/m^2^)	23.0	(3.62)	24.1	(3.47)	22.3	(3.56)
HbA1c	(%)	5.7	(0.62)	5.7	(0.69)	5.7	(0.56)
Glycoalbumin	(%)	14.6	(1.96)	14.4	(2.33)	14.7	(1.64)
Blood glucose	(mg/dL)	96.0	(16.26)	99.3	(17.54)	93.7	(14.91)
Blood insulin	(μU/mL)	5.1	(2.79)	5.1	(3.09)	5.1	(2.56)
HOMA IR	(-)	1.2	(0.83)	1.3	(0.92)	1.2	(0.76)
Triglyceride	(mg/dL)	97.7	(85.08)	124.6	(116.91)	79.1	(44.39)
Total cholesterol	(mg/dL)	204.5	(34.39)	201.9	(34.31)	206.3	(34.35)
HDL cholesterol	(mg/dL)	65.1	(16.59)	58.4	(14.76)	69.7	(16.22)
LDL cholesterol	(mg/dL)	116.0	(29.66)	116.2	(29.17)	115.9	(30.02)
ALT	(U/L)	20.8	(13.91)	26.4	(16.96)	16.9	(9.58)
AST	(U/L)	21.8	(7.88)	23.8	(8.42)	20.5	(7.17)
γ-GTP	(U/L)	32.5	(39.62)	48.0	(55.19)	21.8	(16.47)
Creatinine	(mg/dL)	0.7	(0.52)	0.9	(0.70)	0.6	(0.31)
Urea nitrogen	(mg/dL)	14.5	(4.54)	15.4	(4.51)	13.9	(4.46)
Plasma pentosidine	(pmol/mL)	26.6	(16.09)	26.2	(18.66)	26.9	(14.04)
SBP	(mmHg)	120.8	(16.96)	123.9	(16.86)	118.7	(16.71)
DBP	(mmHg)	76.9	(11.36)	79.6	(11.54)	75.0	(10.83)
Physical functioning	(points)	90.3	(15.31)	92.1	(14.02)	89.1	(16.04)
Role physical	(points)	90.6	(17.75)	92.7	(16.43)	89.2	(18.48)
Bodily pain	(points)	73.3	(22.76)	76.0	(22.57)	71.4	(22.71)
General health	(points)	61.3	(17.55)	62.3	(17.07)	60.6	(17.85)
Vitality	(points)	62.3	(19.08)	65.0	(19.15)	60.3	(18.81)
Social functioning	(points)	91.6	(16.08)	93.0	(15.12)	90.6	(16.65)
Role emotional	(points)	91.8	(17.32)	92.6	(17.27)	91.4	(17.34)
Mental health	(points)	75.3	(17.02)	76.8	(16.88)	74.3	(17.05)
**Categorical variables**		**n**	**(%)**	**n**	**(%)**	**n**	**(%)**
Diabetes mellitus	No	988	(93.9%)	399	(92.8%)	589	(94.7%)
	Yes	64	(6.1%)	31	(7.2%)	33	(5.3%)
Hyperlipidemia	No	872	(83.0%)	354	(82.7%)	518	(83.3%)
	Yes	178	(17.0%)	74	(17.3%)	104	(16.7%)
High blood pressure	No	788	(74.8%)	301	(70.0%)	487	(78.2%)
	Yes	265	(25.2%)	129	(30.0%)	136	(21.8%)
Antihypertensive medication use	No	807	(76.6%)	313	(72.8%)	494	(79.3%)
	Yes	246	(23.4%)	117	(27.2%)	129	(20.7%)
Exercising (non-winter seasons)	No	819	(77.9%)	334	(77.9%)	485	(77.8%)
	Yes	233	(22.1%)	95	(22.1%)	138	(22.2%)
Exercising (winter season)	No	817	(78.1%)	336	(78.7%)	481	(77.7%)
	Yes	229	(21.9%)	91	(21.3%)	138	(22.3%)
Smoking	No	669	(64.0%)	181	(42.5%)	488	(78.7%)
	Current	180	(17.2%)	125	(29.3%)	55	(8.9%)
	Previous	197	(18.8%)	120	(28.2%)	77	(12.4%)
Alcohol consumption	No	501	(48.1%)	121	(28.3%)	380	(61.8%)
	Current	499	(47.9%)	293	(68.6%)	206	(33.5%)
	Previous	42	(4.0%)	13	(3.0%)	29	(4.7%)

BMI, body mass index; HbA1c, hemoglobin A1c; HOMA-IR, homeostatic model assessment-estimated insulin resistance; HDL, high-density cholesterol; LDL, low-density cholesterol; ALT, alanine transaminase; AST, aspartate transaminase; γ-GTP, γ-glutamyl transferase; SBP, systolic blood pressure; DBP, diastolic blood pressure; SD, standard deviation.

**Table 2:** Correlation between SF-36 scores and blood glucose levels (mg/dL).

Characteristics	Univariate					Model 1					Model 2				
	β	95% CI			*p*-Value	β	95% CI			*p*-Value	β	95% CI			*p*-Value
Physical functioning	−0.153	−0.209	to	−0.097	< 0.001	0.012	−0.044	to	0.068	0.671	0.019	−0.037	to	0.075	0.499
Role physical	−0.101	−0.167	to	−0.035	0.003	0.002	−0.068	to	0.072	0.958	0.015	−0.057	to	0.087	0.685
Bodily pain	−0.117	−0.201	to	−0.032	0.007	−0.015	−0.107	to	0.078	0.756	0.009	−0.084	to	0.103	0.847
General health	−0.140	−0.205	to	−0.075	< 0.001	−0.107	−0.179	to	−0.034	0.004	−0.081	−0.154	to	−0.008	0.030
Vitality	0.063	−0.008	to	0.134	0.080	−0.018	−0.096	to	0.061	0.661	−0.014	−0.093	to	0.066	0.736
Social functioning	0.001	−0.059	to	0.061	0.982	0.001	−0.066	to	0.068	0.972	0.005	−0.064	to	0.074	0.891
Role emotional	−0.005	−0.070	to	0.059	0.875	0.031	−0.041	to	0.104	0.397	0.029	−0.045	to	0.103	0.438
Mental health	0.030	−0.033	to	0.094	0.345	−0.019	−0.090	to	0.052	0.603	−0.022	−0.094	to	0.050	0.544

Model 1: adjusted for age, sex, and BMI. Model 2: adjusted for age, sex, BMI, antihypertensive medication, exercise (non-winter and winter seasons), smoking, and alcohol consumption. BMI, body mass index; CI, confidence interval; SF-36, Short Form Health Survey 36.

**Table 3:** Correlation between SF-36 scores and HOMA-IR values.

Characteristics	Univariate					Model 1					Model 2				
	β	95% CI			*p*-Value	β	95% CI			*p*-Value	β	95% CI			*p*-Value
Physical functioning	−1.795	−2.907	to	−0.684	0.002	0.373	−0.786	to	1.532	0.528	0.719	−0.455	to	1.893	0.230
Role physical	−0.913	−2.206	to	0.380	0.166	−0.040	−1.499	to	1.419	0.957	0.330	−1.184	to	1.844	0.669
Bodily pain	−1.431	−3.088	to	0.226	0.090	0.561	−1.359	to	2.481	0.566	0.769	−1.197	to	2.734	0.443
General health	−3.503	−4.765	to	−2.241	< 0.001	−3.328	−4.824	to	−1.832	< 0.001	−2.646	−4.181	to	−1.111	0.001
Vitality	−0.135	−1.526	to	1.256	0.849	−0.269	−1.893	to	1.355	0.745	0.116	−1.558	to	1.791	0.892
Social functioning	−0.961	−2.132	to	0.210	0.107	−0.470	−1.861	to	0.920	0.507	−0.548	−1.991	to	0.895	0.456
Role emotional	−1.134	−2.395	to	0.126	0.078	−0.968	−2.466	to	0.531	0.206	−0.783	−2.333	to	0.768	0.322
Mental health	−0.196	−1.437	to	1.045	0.756	−0.468	−1.936	to	1.000	0.532	−0.387	−1.901	to	1.126	0.616

Model 1: adjusted for age, sex, and BMI. Model 2: adjusted for age, sex, BMI, antihypertensive medication, exercise (non-winter and winter seasons), smoking, and alcohol consumption. BMI, body mass index; CI, confidence interval; HOMA-IR, homeostatic model assessment-estimated insulin resistance; SF-36, Short Form Health Survey 36.

**Table 4:** Correlation between SF-36 scores and HbA1c levels (%).

Characteristics	Univariate					Model 1					Model 2				
	β	95% CI			*p*-Value	β	95% CI			*p*-Value	β	95% CI			*p*-Value
Physical functioning	−4.859	−6.333	to	−3.385	< 0.001	−0.728	−2.156	to	0.701	0.318	−0.427	−1.853	to	1.000	0.557
Role physical	−4.249	−5.972	to	−2.527	< 0.001	−1.715	−3.511	to	0.081	0.061	−1.320	−3.156	to	0.516	0.159
Bodily pain	−3.418	−5.642	to	−1.195	0.003	−0.505	−2.872	to	1.862	0.676	−0.060	−2.448	to	2.327	0.961
General health	−4.728	−6.426	to	−3.030	< 0.001	−3.824	−5.670	to	−1.977	< 0.001	−3.321	−5.184	to	−1.458	< 0.001
Vitality	−0.142	−2.014	to	1.730	0.882	−1.894	−3.892	to	0.105	0.063	−1.636	−3.666	to	0.395	0.114
Social functioning	−1.325	−2.901	to	0.250	0.099	−1.299	−3.012	to	0.414	0.137	−1.058	−2.810	to	0.693	0.236
Role emotional	−1.206	−2.904	to	0.492	0.164	−0.438	−2.287	to	1.412	0.643	−0.334	−2.217	to	1.550	0.728
Mental health	−0.008	−1.678	to	1.662	0.992	−1.055	−2.864	to	0.754	0.253	−1.130	−2.967	to	0.706	0.227

Model 1: adjusted for age, sex, and BMI. Model 2: adjusted for age, sex, BMI, antihypertensive medication, exercise (non-winter and winter seasons), smoking, and alcohol consumption. BMI, body mass index; CI, confidence interval; SF-36, Short Form Health Survey 36.

**Table 5:** Correlation between SF-36 scores and glycoalbumin levels (%).

Characteristics	Univariate					Model 1					Model 2				
	β	95% CI			*p*-Value	β	95% CI			*p*-Value	β	95% CI			*p*-Value
Physical functioning	−1.482	−1.948	to	−1.017	< 0.001	−0.374	−0.813	to	0.065	0.095	−0.290	−0.725	to	0.145	0.191
Role physical	−1.543	−2.084	to	−1.002	< 0.001	−0.659	−1.210	to	−0.107	0.019	−0.536	−1.096	to	0.025	0.061
Bodily pain	−1.062	−1.763	to	−0.360	0.003	−0.341	−1.069	to	0.387	0.359	−0.212	−0.941	to	0.517	0.569
General health	−1.226	−1.764	to	−0.687	< 0.001	−1.057	−1.626	to	−0.488	< 0.001	−0.883	−1.453	to	−0.313	0.002
Vitality	0.041	−0.550	to	0.631	0.893	−0.447	−1.063	to	0.168	0.154	−0.351	−0.972	to	0.270	0.268
Social functioning	−0.222	−0.719	to	0.276	0.382	−0.295	−0.822	to	0.232	0.272	−0.232	−0.767	to	0.303	0.395
Role emotional	−0.474	−1.009	to	0.061	0.083	−0.276	−0.845	to	0.292	0.341	−0.227	−0.803	to	0.348	0.438
Mental health	−0.057	−0.584	to	0.470	0.831	−0.339	−0.895	to	0.218	0.233	−0.323	−0.885	to	0.238	0.258

Model 1: adjusted for age, sex, and BMI. Model 2: adjusted for age, sex, BMI, antihypertensive medication, exercise (non-winter and winter seasons), smoking, and alcohol consumption. BMI, body mass index; CI, confidence interval; SF-36, Short Form Health Survey 36.

**Table 6:** Correlation between SF-36 scores and pentosidine levels (pmol/mL).

Characteristics	Univariate					Model 1					Model 2				
	β	95% CI			*p*-Value	β	95% CI			*p*-Value	β	95% CI			*p*-Value
Physical functioning	−0.143	−0.200	to	−0.086	< 0.001	−0.040	−0.093	to	0.012	0.132	−0.018	−0.073	to	0.037	0.516
Role physical	−0.191	−0.256	to	−0.125	< 0.001	−0.109	−0.175	to	−0.044	0.001	−0.112	−0.182	to	−0.042	0.002
Bodily pain	−0.102	−0.187	to	−0.016	0.019	−0.042	−0.129	to	0.045	0.341	−0.037	−0.128	to	0.055	0.431
General health	−0.108	−0.173	to	−0.042	0.001	−0.094	−0.162	to	−0.025	0.007	−0.093	−0.164	to	−0.021	0.011
Vitality	0.037	−0.035	to	0.109	0.311	−0.019	−0.093	to	0.054	0.608	−0.002	−0.080	to	0.076	0.956
Social functioning	−0.057	−0.117	to	0.004	0.065	−0.075	−0.138	to	−0.012	0.019	−0.073	−0.140	to	−0.006	0.033
Role emotional	−0.114	−0.179	to	−0.049	0.001	−0.103	−0.171	to	−0.035	0.003	−0.073	−0.144	to	−0.001	0.048
Mental health	0.016	−0.048	to	0.080	0.629	−0.015	−0.082	to	0.051	0.656	−0.007	−0.077	to	0.064	0.856

Model 1: adjusted for age, sex, and BMI. Model 2: adjusted for age, sex, BMI, antihypertensive medication, exercise (non-winter and winter seasons), smoking, and alcohol consumption. BMI, body mass index; CI, confidence interval; SF-36, Short Form Health Survey 36.

## Data Availability

The data reported in this study cannot be shared publicly because of ethical concerns. Data are available from the Hirosaki University COI Program Institutional Data Access/Ethics Committee (contact via e-mail: coi@hirosaki-u.ac.jp) for researchers who meet the criteria for access to the data. Researchers need to be approved by the research ethics review board of the organization of their affiliations.

## Supplementary Materials

The following supporting information can be downloaded at: https://www.mdpi.com/article/10.3390/ijerph19159391/s1, Table S1: Correlation between SF-36 scores and age, sex, or BMI.
