# Supplementary material for: Correlation between Glycation-Related Biomarkers and Quality of Life in the General Japanese Population: The Iwaki Cross-Sectional Research Study
Source: Int J Environ Res Public Health. 2022 Jul 31;19(15):9391. doi: 10.3390/ijerph19159391 (PMC9368172; doi:10.3390/ijerph19159391)
Supplement: Supplementary file 1 [file ijerph-19-09391-s001.zip › ijerph-1775998-supplementary.pdf]

Table S1 : Correlation between SF-36 scores and age, sex, or BMI.

Characteristics	Age			Sex (female)			BMI		
	β	95% CI	*p* -value	β	95% CI	*p* -value	β	95% CI	*p* -value
Physical functionin	-0.446	-0.499 to -0.392	<0.001	-4.006	-5.697 to -2.314	<0.001	-0.628	-0.859 to -0.397	<0.001
Role physical	-0.356	-0.423 to -0.288	<0.001	-3.722	-5.851 to -1.593	0.001	-0.181	-0.472 to 0.110	0.222
Bodily pain	-0.278	-0.367 to -0.189	<0.001	-5.639	-8.442 to -2.835	<0.001	-0.650	-1.032 to -0.267	0.001
General health	-0.112	-0.181 to -0.042	0.002	-2.461	-4.666 to -0.256	0.029	-0.444	-0.745 to -0.143	0.004
Vitality	0.211	0.137 to 0.286	<0.001	-5.024	-7.395 to -2.652	<0.001	-0.160	-0.484 to 0.163	0.332
Social functioning	0.030	-0.035 to 0.094	0.367	-2.968	-4.999 to -0.938	0.004	-0.316	-0.593 to -0.038	0.026
Role emotional	-0.084	-0.153 to -0.015	0.017	-1.441	-3.629 to 0.747	0.197	-0.152	-0.451 to 0.147	0.320
Mental health	0.117	0.050 to 0.185	0.001	-2.592	-4.736 to -0.447	0.018	-0.043	-0.336 to 0.250	0.774

Adjusted for age, sex, and BMI.

BMI, body mass index; CI, confidence interval; SF-36, Short Form Health Survey 36.
